# STENCIL-NET for equation-free forecasting from data

**DOI:** 10.1038/s41598-023-39418-6

**Published:** 2023-08-07

**Authors:** Suryanarayana Maddu, Dominik Sturm, Bevan L. Cheeseman, Christian L. Müller, Ivo F. Sbalzarini

**Affiliations:** 1 Faculty of Computer Science, Technische Universität Dresden, Dresden, Germany; 2 Max Planck Institute of Molecular Cell Biology and Genetics, Dresden, Germany; 3 Center for Systems Biology Dresden, Dresden, Germany; 4 Helmholtz-Zentrum Dresden-Rossendorf (HZDR), Dresden, Germany; 5 Center for Advanced Systems Understanding (CASUS), Görlitz, Germany; 6 Department of Statistics, LMU München, Munich, Germany; 7 Institute of Computational Biology, Helmholtz Zentrum München, Munich, Germany; 8 Center for Computational Mathematics, Flatiron Institute, New York City, USA; 9 Center for Scalable Data Analytics and Artificial Intelligence ScaDS.AI Dresden/Leipzig, Dresden, Germany; 10 Present Address: Center for Computational Biology, Flatiron Institute, New York City, USA; 11 Present Address: ONI, Inc., Linacre House, Banbury Road, Oxford, OX2 8TA UK

**Keywords:** Applied mathematics, Computer science, Computational science

## Abstract

We present an artificial neural network architecture, termed STENCIL-NET, for equation-free forecasting of spatiotemporal dynamics from data. STENCIL-NET works by learning a discrete propagator that is able to reproduce the spatiotemporal dynamics of the training data. This data-driven propagator can then be used to forecast or extrapolate dynamics without needing to know a governing equation. STENCIL-NET does not learn a governing equation, nor an approximation to the data themselves. It instead learns a discrete propagator that reproduces the data. It therefore generalizes well to different dynamics and different grid resolutions. By analogy with classic numerical methods, we show that the discrete forecasting operators learned by STENCIL-NET are numerically stable and accurate for data represented on regular Cartesian grids. A once-trained STENCIL-NET model can be used for equation-free forecasting on larger spatial domains and for longer times than it was trained for, as an autonomous predictor of chaotic dynamics, as a coarse-graining method, and as a data-adaptive de-noising method, as we illustrate in numerical experiments. In all tests, STENCIL-NET generalizes better and is computationally more efficient, both in training and inference, than neural network architectures based on local (CNN) or global (FNO) nonlinear convolutions.

## Introduction

Often in science and engineering, measurement data from a dynamical processes in space and time are available, but a first-principles mathematical model may not be. Numerical simulation methods can then not be used to predict system behavior. This situation is particularly prevalent in areas such as biology, medicine, environmental science, economy, and finance. There has therefore been much interest in using machine-learning models for data-driven forecasting of space-time dynamics with unknown governing equation.

Recent advancements in data-driven forecasting techniques using artificial neural networks include the ODIL framework^1^ and a mesh-free variant using graph neural networks^2^. In addition, the feasibility of data-driven forecasting of chaotic dynamics has been demonstrated using neural networks with recurrent connections^3–5^. The common goal of these approaches is to learn a discrete propagator of the observed dynamics. This propagator is a combination of the values on finitely many discrete sampling points at a certain time *t* that explains the values at the next time point $t+\Delta t$. As such, these approaches neither learn a governing equation of the observed dynamics, like sparse regression methods^6–8^ or PDE-nets^9,10^ do, nor a data-guided approximation of the solution to a known governing equation, like Physics-Informed Neural Networks do^11,12^. Instead, they learn discrete rules that explain the dynamics of the data. This makes equation-free forecasting from data feasible for a number of applications, including prediction, extrapolation, coarse-graining, and de-noising.

The usefulness of data-driven forecasting methods, however, hinges on the accuracy and stability of the propagator they learned. Ideally, the propagator fulfills the constraints for a valid numerical scheme in the discretization used to represent the data. Then, the learned propagators can be expected to possess generalization power. This has been accomplished when the governing equation is known^13^ and for explicit time-integration schemes^14,15^. Moreover, work on solution- and resolution-specific discretization of nonlinear PDEs has shown that Convolutional Neural Network (CNN) filters are able to generalize to larger spatial solution domains than they have been trained on^16^. However, an over-complete set of CNN filters was used, which renders their training computationally expensive and data-demanding. It remains a challenge to achieve data-efficient and self-consistent forecasting for general spatio-temporal dynamics with unknown governing equation that generalizes to coarser grids in order to accelerate prediction.

Here, we present the STENCIL-NET architecture for equation-free forecasting of nonlinear and/or chaotic dynamics from spatiotemporal data across different grid resolutions. STENCIL-NET is inspired by works on learning data-adaptive discretizations for nonlinear PDEs, which have been shown to generalize to coarser grids^17^. This generalization ability derives from the inductive bias gained when a Multi-Layer Perceptron (MLP) architecture is combined with a known consistent time-stepping scheme, such as a Runge-Kutta method^18^ or Total Variation Diminishing (TVD) methods^19,20^. On a regular Cartesian grid, it is straightforward to represent the spatial discretization by a neural network architecture. STENCIL-NET relies on sliding a small MLP over the input patches to perform cascaded cross-channel parametric pooling, which enables learning complex features of the dynamics. We illuminate the mathematical relationship between this neural network architecture and ENO/WENO finite differences^19,20^. This connection to classic numerical methods constrains the propagators learned by STENCIL-NET to be consistent on average in the sense of numerical analysis, i.e., the prediction errors on average decrease for increasing spatial resolution and increasing stencil size. Therefore, STENCIL-NETs can be used to produce predictions beyond the space and time horizons they were trained for, for chaotic dynamics, and for coarser grid resolutions than they were trained for, all of which we show in numerical experiments. We also show that STENCIL-NETs do this better than both Convolutional Neural Networks (CNN) based local nonlinear convolutions and Fourier Neural Operators (FNO), which rely on a combination of global Fourier modes and nonlinear convolutions. As a consequence of their accuracy and stability, STENCIL-NETs extrapolate better to coarser grid resolutions and are computationally more efficient in both training and inference/simulation than CNNs and FNOs with comparable numbers of trainable parameters.

## The STENCIL-NET

Consider the following dynamic process in discrete space and time:

1 $$\begin{aligned} \frac{\partial u_i}{\partial t} = {\mathscr {N}}_d \big ( {\textbf{u}}_m(x_i),\, \Xi , \Delta x \big ), \quad i=1,\ldots ,N_x, \end{aligned}$$

where $u_i = u(x_i,t_j)$ are the $N_x$ data points in space and $N_t$ in time, and $\Xi$ are unknown parameters. The subset ${\textbf{u}}_m(x_i) = \{u(x_j): x_j \in S_m(x_i) \}$ are the $(2m+1)$ data points within some finite stencil support $S_m$ of radius *m* around point $x_i$ at time *t*. $\Delta x$ is the grid resolution of the spatial discretization and ${\mathscr {N}}_d: {\mathbb {R}}^{2m+1} \mapsto {\mathbb {R}}$ is the nonlinear discrete propagator of the data. Integrating Eq. (1) on both sides over one time step $\Delta t$ yields a discrete map from $u(x_i,t)$ to $u(x_i,t+\Delta t)$ as:

2 $$\begin{aligned} u_i^{n+1} = u_i^{n} + \displaystyle \int \limits _{t}^{t+\Delta t} {\mathscr {N}}_d \big ( {\textbf{u}}_m^{n}(x_i), \Xi , \Delta x \big ) \, \textrm{d}\tau , \end{aligned}$$

where $u_{i}^{n+1} = u(x_i,t+\Delta t)$, $u_i^n = u(x_i, t)$, and ${\textbf{u}}_m^n (x_i) = \{u(x_j,t): x_j \in S_m (x_i) \}$. Approximating the integral on the right-hand side by quadrature, we find

3 $$\begin{aligned} u_i^{n+1} = {\textbf{T}}_d \bigg ( u_{i}^n, \, {\mathscr {N}}_d \big ( {\textbf{u}}_m^n(x_i), \Xi , \Delta x \big ), \Delta t\bigg ) + {O}(\Delta t^{r}), \quad n=0,\ldots ,N_t -1, \end{aligned}$$

where $N_t$ is the total number of time steps. Here, ${\textbf{T}}_d$ is the explicit discrete time integrator with time-step size $\Delta t$. Due to approximation of the integral by quadrature, the discrete time integrator converges to the continuous-time map in Eq. (2) with temporal convergence rate *r* as $\Delta t \rightarrow 0$. Popular examples of explicit time-integration schemes include forward Euler, Runge-Kutta, and TVD Runge-Kutta methods. In this work, we only consider Runge-Kutta-type methods and their TVD variants ^20^. STENCIL-NET approximates the discrete nonlinear function ${\mathscr {N}}_d (\cdot )$ with a neural network, leading to:

4 $$\begin{aligned} {\hat{u}}_i^{n+k} = {\textbf{T}}_d^k \bigg ( {\hat{u}}_{i}^n, {\mathscr {N}}_{\theta }\big ( {\textbf{u}}_m^n(x_i), \Xi \big ), \Delta t\bigg ), \end{aligned}$$

where ${\mathscr {N}}_{\theta }: {\mathbb {R}}^{2m+1} \mapsto {\mathbb {R}}$ are the nonlinear network layers with weights $\theta$. The superscript *k* in ${\textbf{T}}_d^k$ denotes that the discrete propagator maps *k* time steps into the future.

Assuming point-wise uncorrelated noise $\eta$ on the data, i.e., $v_i^n = u_i^n + \eta _i^n$, Eq. (4) can be extended for mapping noisy data from $v_i^{n}$ to $v_i^{n+k}$, as:

5 $$\begin{aligned} {\hat{v}}_i^{n+k} = {\textbf{T}}_d^{k} \Big ( {\hat{v}}_i^n - {\hat{\eta }}_i^n, {\mathscr {N}}_{\theta }\big ( {\textbf{v}}_m^n(x_i) - \hat{{\textbf{n}}}_m^n(x_i), \Xi \big ), \Delta t\Big ) + {\hat{\eta }}_i^{n+k}, \end{aligned}$$

where ${\textbf{v}}_m^n(x_i) = \{v(x_j,t): x_j \in S_m (x_i) \}$ are the given noisy data and $\hat{{\textbf{n}}}_m^n(x_i) = \{{\hat{\eta }}(x_j,t): x_j \in S_m (x_i) \}$ the noise estimates on the stencil $S_m$ centered at point $x_i$ at time *t*.

### Neural network architecture

**Figure 1:**
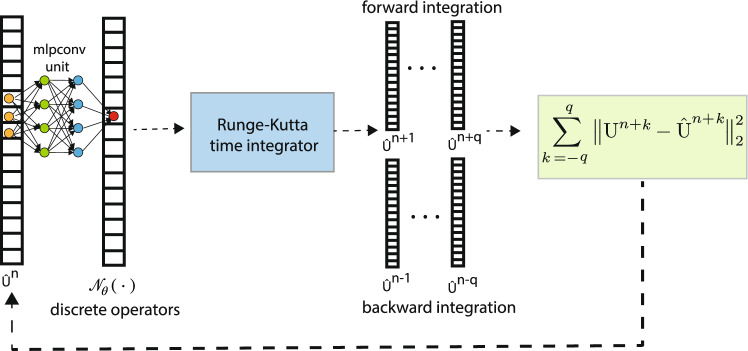
The STENCIL-NET architecture for equation-free forecasting from data. The mlpconv unit performs parametric pooling by moving a small (stencil size $S_m$) MLP network across the input vector $\hat{{\textbf{u}}}^n$ at time *n* to generate the feature maps. The features reaching the output layer are the stencil weights of the discrete propagator ${\mathscr {N}}_{\theta }$. A Runge-Kutta time integrator is used to evolve the network output over time for *q* steps forward and backward in time, which is then used to compute the loss.

STENCIL-NET uses a single MLP convolutional (mlpconv) unit with $N_l$ fully-connected hidden layers inside to represent the discrete propagator ${\mathscr {N}}_{\theta }$ and a discrete Runge-Kutta time integrator for ${\textbf{T}}_d^{k}$. This results in the architecture shown in Fig. 1. Sliding the mlpconv unit over the input state-variable vector ${\textbf{u}}^{n}$ (or ${\hat{\textbf{u}}}^{n}$ during inference) maps the input on the local stencil patch to discretization features. The computation thus performed by the mlpconv unit is:

6 $$\begin{aligned} {\textbf{y}}^1 = \varsigma \left( {\textbf{W}}_1 {\textbf{x}} + {\textbf{b}}_1\right) ,\, \ldots ,\, {\textbf{y}}^{N_l} = \varsigma \left( {\textbf{W}}_{N_l} {\textbf{y}}^{N_l-1} + {\textbf{b}}_{N_l}\right) , \end{aligned}$$

where $\theta = \{ {{\textbf {W}}}_q, {{\textbf {b}}}_q \}_{q=1,2,\ldots , N_l}$ are the trainable weights and biases, respectively, and $\varsigma$ is the (nonlinear) activation function. Usual choices of activation functions include $\tanh$, sigmoid, and ReLU. Sliding the mlpconv unit across the input vector amounts to cascaded cross-channel parametric pooling over a CNN layer ^21^, which allows for trainable interactions across channels for better abstraction of the input data across multiple resolution levels.

This is in contrast to a conventional CNN, where higher-level abstraction is achieved by over-complete sets of filters, at the cost of requiring more training data and incurring extra workload on the downstream layers of the network for composing features^21^. We therefore provide a direct comparison with a CNN architecture where the feature map is generated by convolving the input ${\textbf{x}}$ followed by a nonlinear activation, i.e.,

7 $$\begin{aligned} {\textbf{y}}^{k} = \varsigma ( {\textbf{W}}_k^m {\textbf{x}} + {\textbf{b}}_k), \end{aligned}$$

where ${\textbf{W}}_k^m$ is a circulant (and not dense, as in Eq. (6) matrix that represents the convolution and depends on the size of the filter $|S_m| = (2m+1)$, and ${\textbf{b}}_k$ is the bias term. For further comparison, we also benchmark the mlpconv STENCIL-NET architecture against the global operator-learning method Fourier Neural Operators (FNO)^22^. The FNO computation is:

8 $$\begin{aligned} {\textbf{y}}^1 = \varsigma \left( {\textbf{W}}_1 {\textbf{x}} + {\textbf{b}}_1 + {\mathscr {F}}^{-1} ( {\textbf{R}}_{1} {\mathscr {F}}\left( {\textbf{x}} ) \right) \right) ,\, \ldots ,\, {\textbf{y}}^{N_l} = \varsigma \left( {\textbf{W}}_{N_l} {\textbf{y}}^{N_l-1} + {\textbf{b}}_{N_l} + {\mathscr {F}}^{-1} ( {\textbf{R}}_{N_\ell } {\mathscr {F}}\left( {\textbf{y}}^{N_l-1} ) \right) \right) , \end{aligned}$$

where $\theta = \{ {{\textbf {W}}}_q, \, {{\textbf {R}}}_q,\, {{\textbf {b}}}_q \}_{q=1,2,\ldots , N_l}$ are the trainable weights and biases, and $\varsigma$ is the (nonlinear) activation function. The operators ${\mathscr {F}}$ and ${\mathscr {F}}^{-1}$ are the forward and inverse Fourier transforms, respectively.


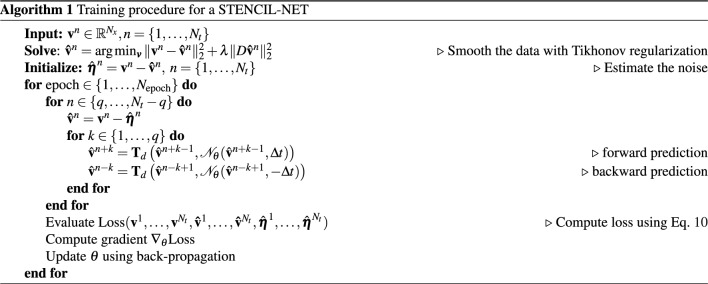


### Loss function and training

From Eq. (5), we derive a loss function for learning the local nonlinear discrete propagator ${\mathscr {N}}_{\theta }$:

9 $$\begin{aligned} {\mathscr {L}}_{MSE} = \sum _{n=1}^{N_t} \sum _{i=1}^{N_x} \sum _{k=-q}^{q} \gamma _k \Big \Vert v_{i}^{n+k} - \bigg ( {\textbf{T}}_d^{k} \Big ( v_i^n - {\hat{\eta }}, {\mathscr {N}}_{\theta }\big ( {\hat{\textbf{v}}}_m^n, \Xi \big ), \Delta t\Big ) + {\hat{\eta }}_i^{n+k} \bigg ) \Big \Vert _{2}^{2}. \end{aligned}$$

The loss compares the forward and backward predictions ${\textbf{T}}_d^{k}$ of the STENCIL-NET with the training data $v_{i}^{n+k}$. It is computed as detailed in Algorithm 1. The positive integer *q* is the number of Runge-Kutta integration steps considered during optimization, which we refer to as the *training time horizon*. The scalars $\gamma _k$ are exponentially decaying (over the training time horizon *q*) weights that account for the accumulating prediction error^23^. In 1D, we treat the noise estimates ${\hat{\eta }}_i$ as latent variables that enable STENCIL-NET to separate dynamics from noise. In that case, we initialize with estimates obtained from Tikonov smoothing of the training data (initialization step in Algorithm 1). For higher-dimensional problems, however, learning noise as a latent variable becomes infeasible. We then instead directly learn a smooth (in time) propagator from the noisy data, as demonstrated in Section "STENCIL-NET for learning smooth dynamics from noisy data".

We penalize the noise estimates $\hat{{\textbf{N}}} = [ \hat{{\textbf{n}}}_m^n ]_{\forall (m,n)}$ in order to avoid learning the trivial solution ${v}_i^{n+k} = {\hat{v}}_i^{n} + {\hat{\eta }}_i^{n+k}$ of the minimization problem in Eq. (9), where ${\mathscr {N}}_\theta \equiv 0$ and only the noise accounts for the data. We also impose a penalty on the weights of the network $\{ {{\textbf {W}}}_i \}_{i=1,2,\ldots , N_l}$ in order to prevent over-fitting. The total loss then becomes:

10 $$\begin{aligned} \text {Loss} = {\mathscr {L}}_{MSE} + \lambda _n \Vert \hat{{{\textbf {N}}}}\Vert _F^2 + \lambda _{wd} \sum _{i=1}^{N_l} \Vert {\textbf{W}}_i \Vert _F^2, \end{aligned}$$

where $N_l$ is the number of layers in the single mlpconv unit, $\hat{{\textbf{N}}} \in {\mathbb {R}}^{N_x \times N_t}$ is the matrix of point-wise noise estimates, and $\Vert \cdot \Vert _F$ is the Frobenius norm of a matrix. We perform grid search through hyper-parameter space in order to identify values of the penalty parameters. We find the choice $\lambda _n = 10^{-5}$ and $\lambda _{wd} = 10^{-8}$ to work well for all problems considered in this paper. Alternatively, methods like drop-out, early-stopping criteria, and Jacobi regularization can be used to counter the over-fitting problem. As a data-augmentation strategy, we unroll the training data both forward and backward in time when evaluating the loss in Eq. (9). Numerical experiments (not shown) confirm that this training mode on average leads to more stable and more accurate models than trained solely forward in time. Regardless, inference is done only forward in time.

Training is done in full-batch mode using an Adam optimizer^24^ with learning rate $lr = 0.001$. As activation functions, we use Exponential Linear Units (ELU) for their smooth function interpolation abilities. While one could readily use ReLU, Leaky-ReLU, $\tanh$, or adaptive activation functions with learnable hyper-parameters, we find that ELU consistently performs best in the benchmark problems considered below. The complete training procedure is summarized in Algorithm 1. To generate a similar CNN architecture for comparison, we replace the mlpconv unit in Fig. 1 with the local nonlinear convolution map from Eq. (7). Similarly, for comparison with FNO, we replace the mlpconv unit with the feature map from Eq. (8) in order to approximate discrete operators through convolutions in frequency space.

## Forecasting accuracy

In classic numerical analysis, stability and accuracy (connected to consistency via the Lax Equivalence Theorem) play central roles is determining the validity of a discretization scheme. In a data-driven setting, learning a propagator of the values on the grid nodes from a time *t* to a later time $t+\Delta t$ assumes that the discrete stencil of the propagator is a valid numerical discretization of some (unknown) ground-truth dynamical system. In this section, we therefore rationalize our choice of network architecture by algebraic parallels with known grid-based discretization schemes. Specifically, we analyze the approximation properties of the STENCIL-NET architecture and argue accuracy by analogy with the well-known class of solution-adaptive ENO/WENO finite-difference schemes^19,20^. As we show below, WENO schemes involve reciprocal functions, absolute values, and “switch statements”, and they are rational functions in the stencil values.

**Figure 2:**
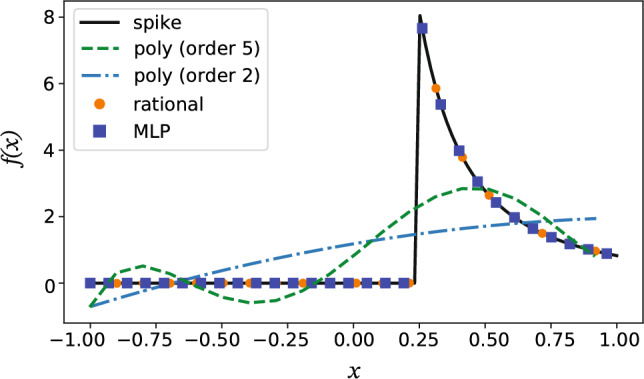
Approximation properties for sharply varying functions. Best rational, polynomial, and MLP fits to the spike function $f(x) =(a + a \vert x - b \vert / (x - b)) (1/(x + c)^2)$ with $a = 0.5$, $b = 0.25$, and $c = 0.1$. The rational function fit uses Newman polynomials^25^ to approximate $\vert x\vert$.

MLPs are particularly effective in representing rational functions ^26^. As a consequence, MLPs can efficiently represent WENO-like stencils when approximating the nonlinear discrete spatial propagator ${\mathscr {N}}_d$. To illustrate this, we compare the best polynomial, rational, and MLP fits to the spike function in Fig. 2. The rational and MLP approximations both closely follow the true spike (black solid line), whereas polynomials fail to capture the “switch statement”, i.e., the division operation that are quintessential for resolving sharp functions.

**Figure 3:**
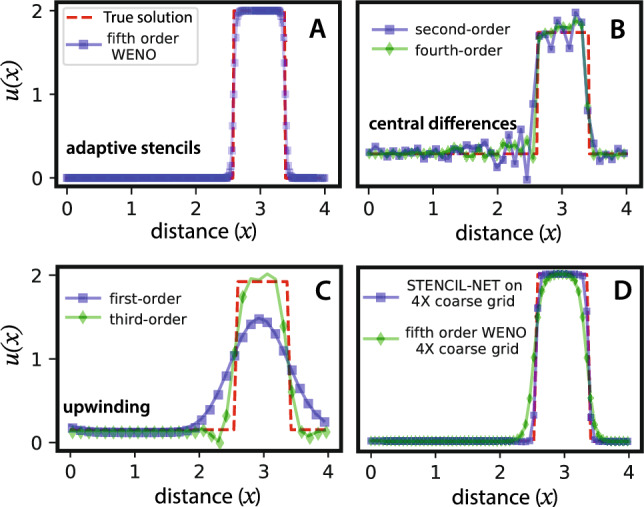
Numerical solutions of advection of a sharp pulse (red dashed line) using different discretization schemes. (**A**) Data-adaptive fifth-order WENO stencils; (**B**) second- and fourth-order central finite differences; (**C**) first- and third-order upwinding schemes; (**D**) STENCIL-NET on a $4\times$ coarser grid compared with fifth-order WENO on the same grid. In all plots the advection velocity is $c=2$ in the one-dimensional advection equation $u_t + cu_x = 0$. All plots are at time $t=3$.

This also explains the results shown in Fig. 3, where the WENO and STENCIL-NET (i.e., MLP) methods accurately resolve the advection of a sharp pulse. Remarkably, the STENCIL-NET can advect the pulse on $4\times$ coarser grids than the WENO scheme is able to (Fig. 3D). All polynomial approximations using central-difference or fixed-stencil upwinding schemes fail to faithfully forecast the dynamics. Based on this empirical evidence, we posit that a relationship between the neural network architecture used and a known class of numerical methods is beneficial for equation-free forecasting, in particular in the presence of sharp gradients or multi-scale features. We therefore next analyze the numerical-method equivalence of the STENCIL-NET architecture.

### Relation with finite-difference stencils

The *l*th spatial derivative at location $x_i$ on a grid with spacing $\Delta x$ can be approximated with convergence order *r* using linear convolutions:

11 $$\begin{aligned} \left. \frac{\partial ^{l} u}{\partial x^{l}}\right| _{x=x_i} = \sum _{x_j \in S_m(x_i)} \!\!\! \xi _j u_j + {O}(\Delta x^r)\,, \end{aligned}$$

where $u_j = u(x_j)$. The $\xi _j$ are the stencil weights that can be determined by local polynomial interpolation^20,27^. The stencil of radius *m* is $S_m(x_i) = \{ x_{i-m}, x_{i-m+1},\ldots ,x_i,\ldots , x_{i+m-1}, x_{i+m} \}$ with size $\vert S_m \vert = 2m+1$. For a spatial domain of size *L*, the spacing is $\Delta x = L/N_{x}$, where $N_x$ is the number of grid points discretizing space. The following propositions from^9,28^ define discrete moment conditions that need to be fulfilled in order for the stencil to be consistent for linear and quadratic operators, respectively:

#### Proposition 3.1

(**Nonlinear convolutions**^9^) Nonlinear terms of order 2, including products of derivatives (e.g., $u u_x, u^2, u_x u_{xx}$) can be approximated by nonlinear convolution. For $l_1,l_2, r_1, r_2 \in {\mathbb {Z}}_{0}^{+}$, we can write,

12 $$\begin{aligned} \left. \left( \frac{\partial ^{l_1} u}{\partial x^{l_1}} \right) \left( \frac{\partial ^{l_2} u}{\partial x^{l_2}} \right) \right| _{x=x_i} = \sum _{x_{j} \in S_{m}(x_i) } \sum _{x_{k} \in S_{m}(x_i)} \xi _{jk} u_j u_k + {O}(\Delta x^{r_1 + r_2})\,, \end{aligned}$$

such that the stencil size $\vert S_m \vert \ge \vert l_1 + r_1 - 1\vert$ and $\vert S_m \vert \ge \vert l_2 + r_2 - 1\vert$. Eq. (12) is a convolution with a Volterra quadratic form, as described in Ref.^29^.

The linear convolutions in Eq. (11) and the nonlinear convolutions in Eq. (12) are based on local polynomial interpolation. They are thus useful to discretize smooth solutions arising in problems like reaction-diffusion, fluid flow at low Reynolds numbers, and elliptic PDEs. But they fail to discretize nonlinear flux terms arising, e.g., in problems like advection-dominated flows, Euler equations, multi-phase flows, level-set and Hamilton-Jacobi equations ^19^. This is because higher-order interpolation near sharp gradients leads to oscillations that do not decay when refining the grid, see Fig. 3B, a fact known as “Gibbs phenomenon”.

Moreover, fixed stencil weights computed from moment conditions can fail to capture the direction of information flow in the data (“upwinding”), leading to non-causal and hence unstable predictions^30,31^. This can be relaxed by biasing the stencil along the direction of information flow or by constructing weights with smooth flux-splitting methods, like Godunov^32^ and Lax-Friedrichs^33^ schemes. To counter the issue of spurious oscillations, artificial viscosity ^34^ can also be added at the cost of lower solution accuracy. Alternatively, data-adaptive stencils with ENO (Essentially Non-Oscillatory)^19^ or WENO (Weighted ENO)^20^ weights are available, which seems the correct choice for data-driven forecasting since they do not only adhere to some moment conditions, but also adapt to the data themselves.

**Figure 4:**
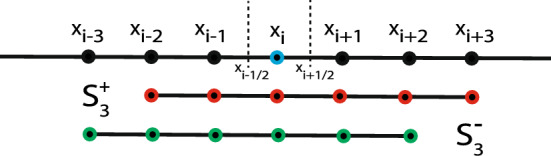
Illustration of data-adaptive stencils. Schematic of the stencil $S_3(x_i)$ centered around the point $x_i$. Here, $S_{3}(x_i) = S_3^{+} \cup S_3^{-}$.

The ENO/WENO method for discretely approximating a continuous function *f* at a point $x_{i \pm 1/2}$ can be written as a linear convolution:

13 $$\begin{aligned} {\hat{u}}_{i\pm {1/2}} = \sum _{x_j \in S^{\pm }(x_i)} \nu _j^{\pm } u(x_j) + {O}(\Delta x^r), \end{aligned}$$

where $u_{i\pm 1/2} = u(x_i \pm \Delta x/2)$, and the function values and coefficients on the stencils are stored in ${\textbf{u}}_m = \{u(x_j): x_j \in S_m(x_i)\}$ and $\nu (x_j)$, respectively. The stencils $S_m^{\pm } (x_i)$ are $S_m^{\pm }(x_i) = S_m(x_i) \setminus x_{i\pm m}$, as illustrated in Fig. 4 for the example of $m=3$. Unlike the fixed-weight convolutions in Eqs. (11) and (12), the coefficients $\nu$ in ENO/WENO stencils are computed based on local smoothness features as approximated on smaller sub-stencils, which in turn depend on the functions values. This leads to locally data-adaptive stencil weights and allows accurate and consistent approximations even if the data $u_i$ are highly varying.

The key idea behind data-adaptive stencils is to use a nonlinear map to choose the locally smoothest stencil, while discontinuities are avoided, to result in smooth and essentially non-oscillatory solutions. The WENO-approximated function values ${\hat{u}}_{i\pm {1/2}}$ from Eq. (13) can be used to approximate spatial variation in the data as follows:

14 $$\begin{aligned} \left. \frac{\partial u}{\partial x} \right| _{x=x_i} = \frac{{\hat{u}}_{i+1/2} - {\hat{u}}_{i-1/2}}{\Delta x} + O\left( \Delta x^r\right) . \end{aligned}$$

In finite-difference methods, the function ${\hat{u}}$ is a polynomial flux, e.g. ${\hat{u}}(u) = c_1 u^2 + c_2 u$, whereas in finite-volume methods, the function *f* is the grid-cell average ${\hat{u}} = \frac{1}{\Delta x} \!\int _{x-\Delta x/2}^{x+\Delta x/2} u(\xi ) \textrm{d}\xi$.

It is clear from Eqs. (11) and (12) that polynomials (i.e., nonlinear convolutions) cannot approximate division operations. Thus, methods using fixed, data-independent stencil weights or multiplications of filters^10^ fail to approximate dynamics with sharp variations. However, as shown in Fig. 2, this can be achieved by rational functions. It is straightforward to see that ENO/WENO stencils are rational functions in the stencil values ${\textbf{u}}_m$. Indeed, from Eq. (13), the stencil weights are computed as $\nu = \frac{{\textbf{g}}_1({\textbf{u}}_m (x_i))}{{\textbf{g}}_2({\textbf{u}}_m (x_i))}$, with a polynomial ${\textbf{g}}_1:{\mathbb {R}}^{2m+1} \rightarrow {\mathbb {R}}$ and a strictly positive polynomial ${\textbf{g}}_2:{\mathbb {R}}^{2m+1} \rightarrow {\mathbb {R}}$ ^20^.

In summary, depending on the smoothness of the data $u_i$, the propagator can either be approximated using fixed stencils that are polynomials in the stencil values (Eqs. 11, 12) or using solution-adaptive stencils that are rational functions in the stencil values (Eqs. (13), (14)). STENCIL-NET can represent either. This leads to the conclusion that any continuous dynamics, smooth or not, ${\mathscr {N}} \left( \cdot \right)$ can be approximated by a polynomial or rational function in local stencil values ${\textbf{u}}_m(x_i)$, up to any desired order of accuracy *r* on a Cartesian grid with resolution $\Delta x$, thus:

15 $$\begin{aligned} {\mathscr {N}}_d \big ( {\textbf{u}}_m(x_i), \Xi \big ) = {\mathscr {N}}({\textbf{u}}({\textbf{x}}), \Xi ) + {O}(\Delta x^r) \text {~~for~~} {\textbf{u}}_m(x_i) = \{ u(x_j): x_j \in S_m(x_i) \}, \end{aligned}$$

where ${\mathscr {N}}_d (\cdot )$ is the discrete propagator.

## Numerical experiments

We apply the STENCIL-NET for learning stable and accurate equation-free forecasting operators for a variety of nonlinear dynamics. For all problems discussed here, we use a single mlpconv unit with $N_l=3$ hidden layers (not counting the input and output layers), and Exponential Linear Unit (ELU) activation functions. Input to the network are the data ${\textbf{u}}_m$ on a stencil of radius $m=3$ in all examples. We present numerical experiments that demonstrate distinct applications of the STENCIL-NET architecture using data from deterministic dynamics, data from chaotic dynamics, and noisy data.

### STENCIL-NET for equation-free forecasting

We first demonstrate the capability of STENCIL-NET to extrapolate space-time dynamics beyond the training domain and to different parameter regimes. For this, we consider the forced Burgers equation in one spatial dimension and time. The forced Burgers equation with a nonlinear forcing term can produce rich and sharply varying solutions. Moreover, we choose the forcing term at random in order to explore generalization to different parts of the solution manifold^16^. The forced Burgers equation in 1D is:

16 $$\begin{aligned} \frac{\partial u}{\partial t} + \frac{\partial (u^2)}{\partial x} = D \frac{\partial ^2 u}{\partial x^2} + f(x,t) \end{aligned}$$

for the unknown function *u*(*x*, *t*) with diffusion constant $D=0.02$. Here, we use the forcing term

17 $$\begin{aligned} f(x,t) = \sum _{i=1}^{N} A_i \sin (\omega _i t + 2\pi l_i x/L + \phi _i) \end{aligned}$$

with each parameter drawn independently and uniformly at random from its respective range: $A \in [ -0.1,0.1 ]$, $\omega \in [-0.4,0.4]$, $\phi \in [0, 2\pi ]$, and $N=20$. The domain size *L* is set to $2\pi$ (i.e., $x\in [0,2\pi ]$) with periodic boundary conditions and $l_i \in \{2,3,4,5\}$. We use a smooth initial condition $u(x,t=0) = \exp ({-(x-3)^2})$ and generate data on $N_x = 256$ evenly spaced grid points with fifth-order WENO discretization of the convection term and second-order central differences for the diffusion term. Time integration is performed using a third-order Runge-Kutta method with time-step size $\Delta t$ chosen as large as possible according to the Courant-Friedrichs-Lewy (CFL) condition of this equation. For larger domains, we adjust the range of $l_i$ so as to preserve the wavenumber spectrum of the dynamics, e.g., for $L = 8\pi$ we use $l_i \in \{8,9,\ldots ,40\}$. We use the same spatial resolution $\Delta x$ for all domain sizes, i.e., the total number of grid points $N_x$ grows proportionally to domain size.

We train STENCIL-NET at different spatial resolutions $\Delta x_c = (C\Delta x)$, where *C* is the sub-sampling factor. We use sub-sampling factors $C \in \{2,4,8\}$ in space. We sub-sample the data by simply removing intermediate grid points. For example, for $C=2$, we only keep spatial grid points with even indices. During training, time integration is done with a step size that satisfies the CFL condition in the sub-sampled mesh, i.e., $\Delta t_c \le (\Delta x_c)^2/D$, where $\Delta x_c = C\Delta x$ and $\Delta t_c$ are the training space and time resolutions, respectively. Training is done in the full spatial domain of the data for times 0 through 40, independent for each resolution. We then test how well STENCIL-NET is able to forecast the solution to times >40. Due to the steep gradients and rapidly varying dynamics of the Burgers equation, this presents a challenging problem for testing STENCIL-NET’s generalization capabilities.

**Figure 5:**
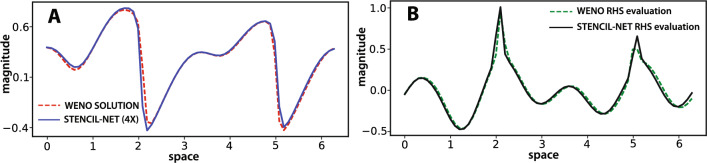
Comparison between STENCIL-NET (4-fold coarsened) and fifth-order WENO for forced Burgers. (**A**) Comparison between the STENCIL-NET prediction and WENO data over the entire domain at time $t= 40$, i.e., at the end of the training time horizon. (**B**) Comparison of the nonlinear discrete operators learned by STENCIL-NET (${\mathscr {N}}_{\theta }$) and the ground-truth WENO scheme (${\mathscr {N}}_{d}$) at time $t=40$.

**Figure 6:**
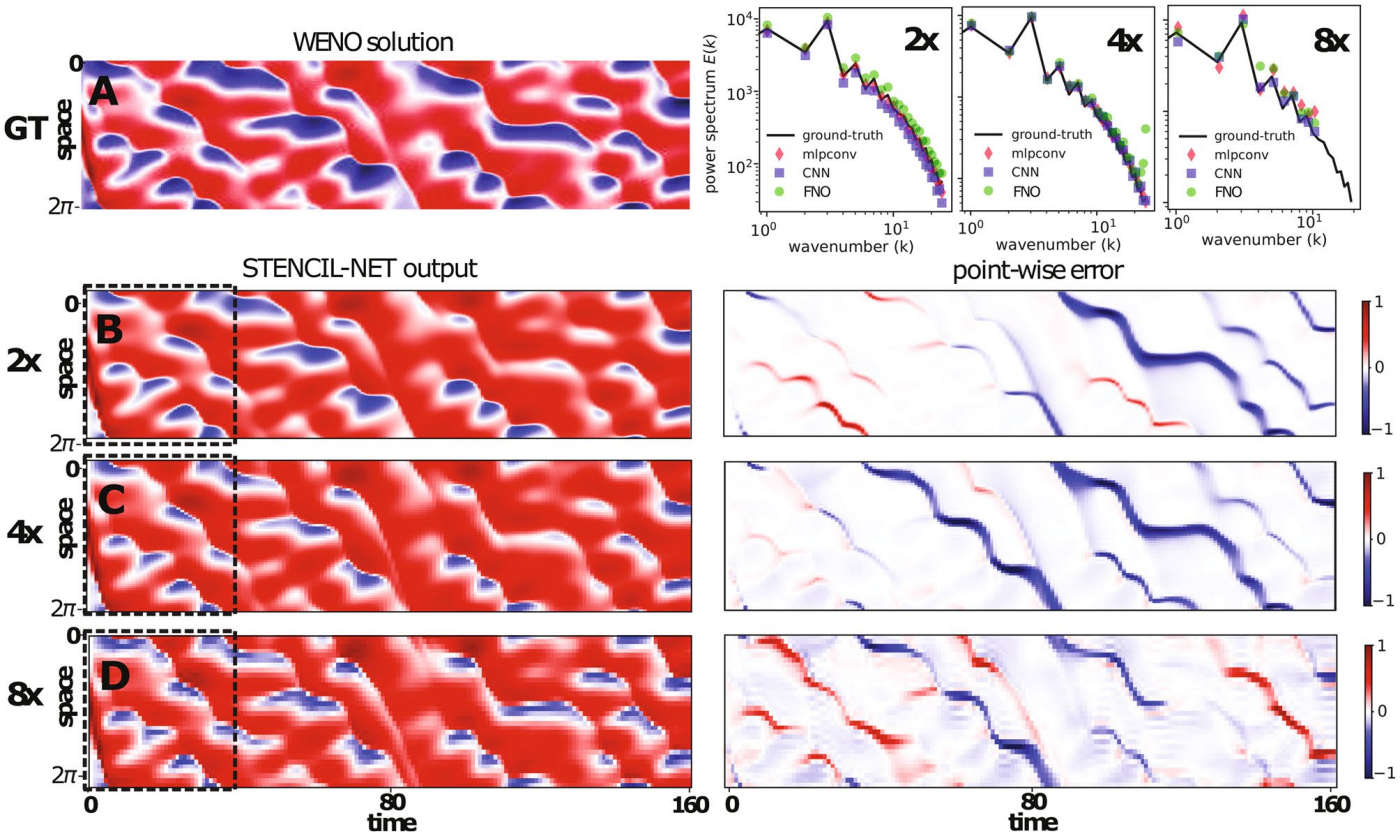
Forced Burgers forecasting with STENCIL-NET on coarser grids. (**A**) Left: fifth-order WENO ground-truth (GT) data with spatial resolution $N_x = 256$. Right: power spectra of the predictions from different architectures (STENCIL-NET, CNN, FNO) compared to ground-truth. (**B**,**C**,**D**) Left column: output of STENCIL-NET on $2\times$, $4\times$, and $8\times$ coarser grids. The dashed boxes contain the data used for training at each resolution. Right column: point-wise absolute error of the STENCIL-NET prediction compared to the ground-truth (GT) data in (**A**) beyond the training domain.

Figure 5A compares the STENCIL-NET prediction on a four-fold downsampled grid (i.e., $C=4$) at the end of the training time interval after training on the full-resolution training data. Figure 5B compares the learned nonlinear discrete propagator ${\mathscr {N}}_\theta$ from the STENCIL-NET with the true discrete ${\mathscr {N}}_d$ of the simulation that generated the data. It is thanks to the good match in this propagator that STENCIL-NET is able to generalize for parameters and domain-sizes beyond the training conditions. Indeed, this is what we observe in Fig. 6, where we compare the fifth-order accurate WENO data on a fine grid $(\Delta x = L/N_x,\, N_x = 256,\, L=2\pi )$ with the STENCIL-NET predictions on coarsened grids with $\Delta x_c = C\Delta x$ for sub-sampling factors $C \in \{2,4,8\}$ and for longer times. STENCIL-NET produces stable and accurate forecasts on all coarser grids for times >40 beyond the training data (dashed black box). The point-wise absolute prediction error (right column) grows when leaving the training time domain, but does not “explode” and is concentrated around the steep gradients in the solution, as expected.

We compare the inference accuracy and computational performance of the mlpconv-based STENCIL-NET with a CNN and with the operator-learning architecture FNO. The CNN relies on local nonlinear convolutions, whereas the FNO learns continuous operators using a combination of nonlinear convolutions and global Fourier modes. Both have previously been proposed for dynamics forecasting from data^22,35^. In all comparisons, we use a CNN with 5 hidden layers of 10 filters each, and a filter radius $m=3$ that matches the stencil radius of the STENCIL-NET. For the FNO, we use 5 hidden layers with 6 local convolutional filters per layer. We rule out spectral bias^36^ by verifying that the fully trained networks can represent the Fourier spectra of the ground-truth at the different sub-samplings tested. The results in Fig. 6 show that all three neural-network approximations are able to capture the power spectrum of the true signal. Next, we compare the mean-square errors of the network predictions at different sub-sampling. The results in Table 1 show that while the FNO performs best for low sub-sampling, the STENCIL-NET has the best generalization power to coarse grids. This is consistent with the expectation that FNO achieves superior accuracy when trained with sufficient data^22^. However, the FNO predictions become increasingly unstable during inference for high sub-sampling.

**﻿Table 1:** Prediction Mean Square Error (MSE) comparison between STENCIL-NET, a Convolutional Neural Network (CNN), and a Fourier Neural Operator (FNO) of comparable sizes (i.e., numbers of trainable parameters) for different sub-sampling of the forced Burgers test case after training for 10,000 epochs with $q=2$.

Prediction MSE	$2 \times$	$4 \times$	$8 \times$
STENCIL-NET (2264 parameters)	0.0419	$\mathbf {0.0392}$	$\mathbf {0.0741}$
CNN (2281 parameters)	0.0411	0.0562	0.0823
FNO (2665 parameters)	$\mathbf {0.0351}$	0.0488	0.0835

All networks are trained on data up to time $T = 40$, while the errors are evaluated for predictions up to time $T=160$. Best performance is highlighted in bold.

**Figure 7:**
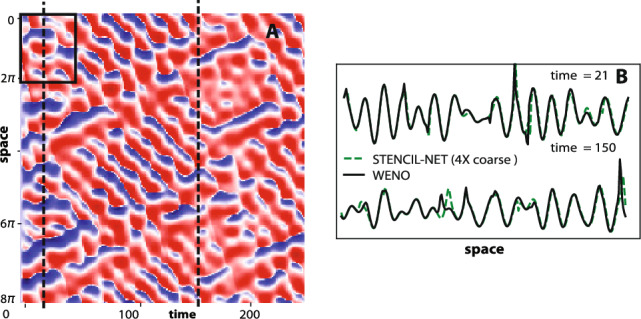
STENCIL-NET extrapolation to larger spatial domains and longer times. (**A**) STENCIL-NET prediction on a $4\times$ coarser grid. (**B**) Comparison between ground-truth discrete propagator ${\mathscr {N}}_d$ (solid) and STENCIL-NET layer output ${\mathscr {N}}_{\theta }$ (dashed) at times 21 (within training data) and 150 (past training data) marked by dashed vertical lines in (**A**). The STENCIL-NET was trained on the data within the domain marked by the solid rectangle in (**A**).

**Figure 8:**
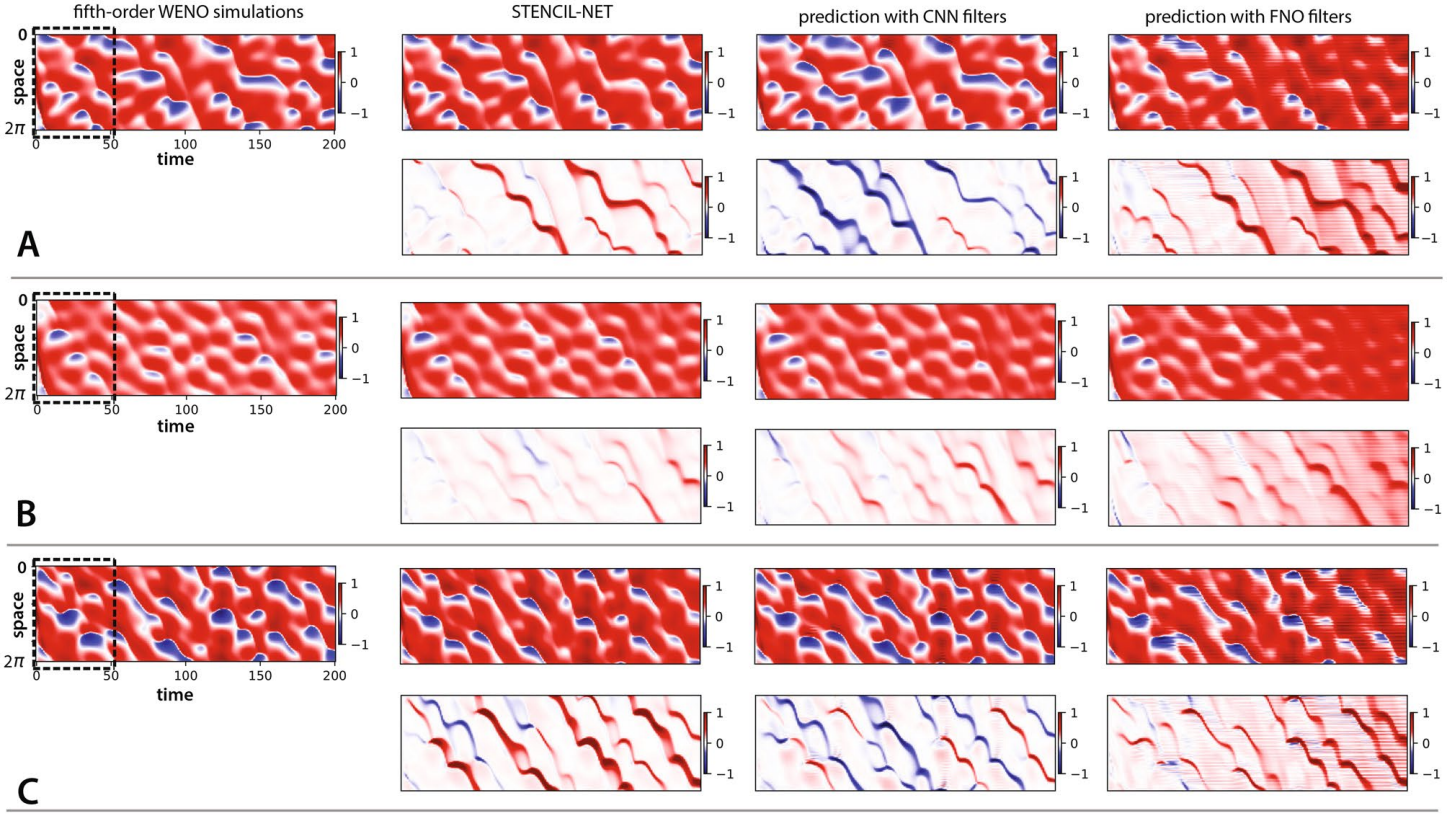
STENCIL-NET generalization to different forcing terms without re-training. Each panel (**A**,**B**,**C**) corresponds to forced Burgers dynamics with a different forcing term (see Eq. 17). Every second row in each panel correspond to point-wise error associated with STENCIL-NET, CNN and FNO (left to right). STENCIL-NET was trained only once using data from (A) (training domain marked by dashed box). Nevertheless, it was able to accurately predict the qualitatively different dynamics for the other forcing terms, since it learned a discrete propagator of the dynamics rather than the solution values themselves. The plots in the first column show the ground-truth data for the different forcing terms, obtained by a fifth-order WENO scheme. The three subsequent columns show the predictions on $4\times$ coarser grids without additional (re-)training and the corresponding absolute errors (w.r.t. WENO) below each plot. The three columns compare STENCIL-NET with a CNN and a FNO of comparable complexity (see Table 1).

In contrast to the generative network proposed in Ref.^14^, which used all time frames for training, the parametric pooling on local stencil patches enables STENCIL-NET to consistently extrapolate also to larger spatial domains, as shown in Fig. 7. Furthermore, as shown in Fig. 8, STENCIL-NET is able to generalize to forcing terms (i.e., dynamics) different from the one used to generate the training data. As seen from the point-wise errors, all architectures perform well within the training window. Also, the largest errors always occurs near steep gradients or jumps in the solution, as expected. The FNO, however, develops spurious oscillations for long prediction intervals.

In addition to its improved generalization power, STENCIL-NET is also computationally more efficient than both the CNN and FNO approaches. This is confirmed in the timings reported in Table 2 for training and inference/simulation, respectively. All times were measured on a Nvidia A100-SXM4 GPU for networks with comparable numbers of trainable parameters.

**Table 2:** Top: Average (over 1000 epochs) training time per epoch compared between the STENCIL-NET, CNN, and FNO from Table 1 with $4\times$ spatial sub-sampling for different training time horizons *q*. Bottom: Average (over 1000 time steps) simulation (inference) time per time-step for the forced Burgers test case with different sub-sampling. Best performance is highlighted in bold as measured on a Nvidia A100-SXM4 GPU.

Training time (sec)	$q=2$	$q =4$	$q=6$
STENCIL-NET	$\mathbf {0.0145}$	$\mathbf {0.0271}$	$\mathbf {0.0398}$
CNN	0.0350	0.0668	0.0989
FNO	0.0664	0.1296	0.1926

Simulation time (sec)	$2 \times$	$4 \times$	$8 \times$
STENCIL-NET	$\mathbf {0.00181}$	$\mathbf {0.00175}$	$\mathbf {0.00172}$
CNN	0.00346	0.00343	0.00336
FNO	0.00692	0.00685	0.00610

**Figure 9:**
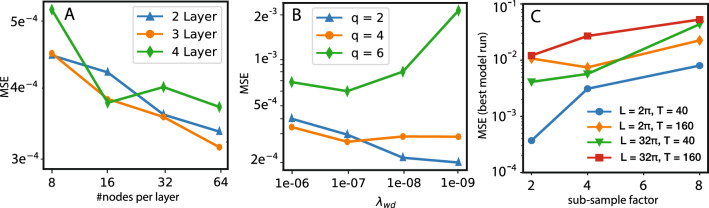
Architecture choice, choice of $\varvec{\lambda _{wd}}$, and generalization power. All models are trained on the spatio-temporal data contained in the dashed box in Fig. 6. The training box encompasses the entire spatial domain of length $L=2\pi$ and a time duration of $T=40$. (**A**) STENCIL-NET prediction accuracy (measured as mean squared error, MSE, w.r.t. ground truth) for different network architectures. Only hidden layers are counted, not the input and output layers. Each data point corresponds to the average MSE accumulated over all stable seed configurations. (**B**) Effect of the weights regularization parameter $\lambda _{wd}$ (see Eq. (10) on the prediction accuracy for different training time horizons *q*. The plots in B were produced with a 3-layer, 64-nodes network architecture, which showed the best performance in A and is used also for all other experiments in this paper. (**C**) Plots to illustrate the generalization power of trained STENCIL-NET to larger domains. Each data point shows the MSE prediction error from the best model run on the grid of the respective resolution (sub-sample factor) for different domain lengths *L* and final times *T*. Training was always done on the data for $L=2\pi$ and $T=40$.

Finally, we analyze the influence of the choice of STENCIL-NET architecture on the results. Figure 9A,B show the effect of the choice of MLP size and of the weight regularization parameter $\lambda _{wd}$ (see Eq. 10) for different training time horizons *q*. Figure 9C compares the accuracies of predictions on grids of varying resolution and for different sizes of the space and time domains (*L* and *T*, respectively). Training was always done for $L=2\pi$, $T=40$. In Figure. 9, we also notice that the prediction error on average decreases for increasing stencil complexity (Fig. 9A) and for increasing mesh resolution (Fig. 9C). This is empirical evidence that STENCIL-NET learns a consistent numerical discretization of the underlying dynamical system.

In summary, we find that a STENCIL-NET model trained on a small training domain can generalize well to longer times, larger domains, and to dynamics containing different forcing terms *f*(*x*, *t*) in Eq. (17) in a numerically consistent way. This highlights the importance of using a network architecture that mathematically relates to numerically valid discrete operators.

### STENCIL-NET as an autonomous predictor of chaotic dynamics

We next analyze how well the generalization power of STENCIL-NET forecasting transfers to inherently chaotic dynamics. Nonlinear dynamical systems, like the Kuramoto-Sivashinsky (KS) model, can describe chaotic spatio-temporal dynamics. The KS equation exhibits varying levels of chaos depending on the bifurcation parameter *L* (domain size) of the system^5^. Given their high sensitivity to numerical errors, KS equations are usually solved using spectral methods. However, data-driven models using Recurrent Neural Networks (RNNs)^5,31,37^ have also shown success in predicting chaotic systems. This is mainly because RNNs are able to capture long-term temporal correlations and implicitly identify the required embedding for forecasting.

**Figure 10:**
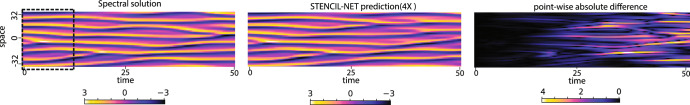
Equation-free forecasting of chaotic Kuramoto-Sivashinsky spatio-temporal dynamics. (Top) Spectral solution of the KS equation on a domain of length $L=64$, where the system behaves chaotically. Data within the dashed rectangle are used for training of the STENCIL-NET model. (Middle) STENCIL-NET forecast on a $4\times$ coarser grid for a $4\times$ longer time. (Bottom) Point-wise absolute difference between the ground-truth data in the top row and the STENCIL-NET forecast in the middle row.

**Figure 11:**
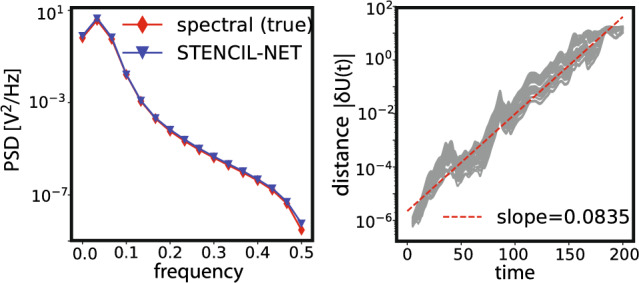
Statistical characteristics of the chaotic system. (Left) Comparison of the power spectral density (PSD) of the ground-truth solution and the STENCIL-NET prediction on a $4\times$ coarser grid. (Right) Growth of the distance between nearby trajectories in the STENCIL-NET prediction, characterizing the maximum Lyaponuv exponent (slope of the dashed red line), compared to the ground truth value $\approx 0.084$ ^38^.

We challenge these results using STENCIL-NET for long-term stable prediction of chaotic dynamics. The training data are obtained from spectral solutions of the KS equation for domain size $L=64$. The KS equation for an unknown function *u*(*x*, *t*) in 1D is:

18 $$\begin{aligned} \frac{\partial u}{\partial t} + \frac{\partial (u^2)}{\partial x} + \frac{\partial ^2 u}{\partial x^2} +\frac{\partial ^4 u}{\partial x^4} = 0. \end{aligned}$$

The domain $x\in [-32,32]$ of length $L=64$ has periodic boundary conditions and is discretized by $N_x = 256$ evenly spaced spatial grid points. We use the initial condition

19 $$\begin{aligned} u(x,t=0) = \sum _{i=1}^{N} A_i \sin (2\pi l_i x/L + \phi _i). \end{aligned}$$

Each parameter is drawn independently and uniformly at random from its respective range: $A \in [ -0.5,0.5 ]$, $\phi \in [0,2\pi ]$, and $l_i \in \{1,2,3\}$. We use a spectral method to numerically solve the KS equation using the chebfun^39^ package. Time integration is performed using a modified exponential time-differencing fourth-order Runge-Kutta method^40^ with step size $\Delta t = 0.05$. For the STENCIL-NET predictions, we use a grid sub-sampling factor of $C=4$ in space, i.e., $N_c=64$, and we train on data up to time 12.5. The prediction time-step size is chosen as large as possible to satisfy the CFL condition.

**Figure 12:**
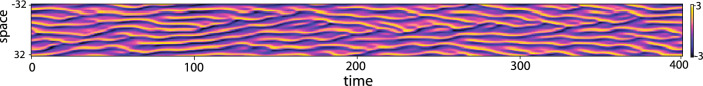
Autonomous prediction of chaotic spatio-temporal dynamics. STENCIL-NET can run as an autonomous predictor of long-term chaotic dynamics with different initial condition and for longer times that it was trained for (training data in the dashed rectangle in Fig. 10) and on a (here $4\times$) coarser grid.

The spatio-temporal dynamic data from the chaotic KS system is shown in Fig. 10. The STENCIL-NET predictions on a $4\times$ coarser grid diverge from the true data over time (see bottom row of Fig. 10). This is due to the chaotic behavior of the dynamics for domain lengths $L>22$, which causes any small prediction error to grow exponentially at a rate proportional to the maximum Lyaponuv exponent of the system. Despite this fundamental unpredictability of the actual space-time values, STENCIL-NET is able to correctly predict the value of the maximum Lyaponuv exponent and the spectral statistics of the system (see Fig. 11). This is evidence that the equation-free STENCIL-NET forecast is consistent in that it has correctly learned the intrinsic ergodic properties of the dynamics that has generated the data. In Fig. 12, we show a STENCIL-NET forecast on a $4\times$ coarser grid for a different initial condition obtained from Eq. (19) with a different random seed, and for longer times ($8\times$) than it was trained for. This shows that the statistically consistent propagator learned by STENCIL-NET can also be run as an autonomous predictor of chaotic dynamics beyond the training domain.

### STENCIL-NET for learning smooth dynamics from noisy data

The discrete time-stepping constraints force any STENCIL-NET prediction to follow a smooth time trajectory. This property can be exploited for filtering true dynamics from noise. We demonstrate this de-noising capability using numerical solution data from the Korteweg-de Vries (KdV) equation with artificially added noise. The KdV equation for an unknown function *u*(*x*, *t*) in 1D is:

20 $$\begin{aligned} \frac{\partial u}{\partial t} + \frac{\partial (u^2)}{\partial x} + \delta \frac{\partial ^3 u}{\partial x^3} = 0\,, \end{aligned}$$

where we use $\delta = 0.0025$. We again use a spectral method to generate numerical data from the KdV equation using the chebfun^39^ package. The domain is $x \in [ -1,1 ]$ with periodic boundary conditions, and the initial condition is $u(x,t=0) = \cos (\pi x)$. The spectral solution is represented on $N_x = 256$ equally spaced grid points discretizing the spatial domain.

**Figure 13:**
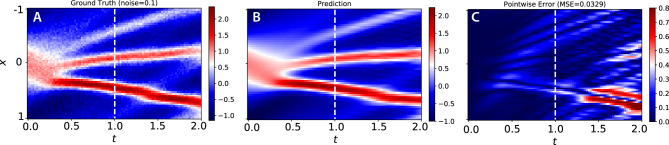
STENCIL-NET for learning smooth dynamics from noisy data. (**A**) Korteweg-de Vries data with point-wise data-dependent additive Gaussian noise of $\sigma =0.1$ used for training. (**B**) STENCIL-NET prediction after training for 10,000 epochs on a $4\times$ coarser grid using data up to time 1.0 (dashed vertical line). (**C**) Point-wise absolute error between the STENCIL-NET prediction and the noise-free ground-truth KdV dynamics.

We corrupt the data vector ${\textbf{U}} = [u(x_i,t_j)]_{\forall (i,j)} \in {\mathbb {R}}^{N_x N_t}$ with element-wise independent additive Gaussian noise:

21 $$\begin{aligned} {\textbf{V}} = {\textbf{U}} + \varvec{\eta }, \end{aligned}$$

with each element of $\varvec{\eta }$, $\eta = \sigma {\mathscr {N}}(0, \text {std}^2({\textbf{U}}))$, where ${\mathscr {N}}(m,V)$ is the normal distribution with mean *m* and variance *V*, and $\text {std}({\textbf{U}})$ is the empirical standard deviation of the data ${\textbf{U}}$, rendering the noise data-dependent. The parameter $\sigma$ is the magnitude of the noise.

We use $\sigma =0.1$ and train a STENCIL-NET over the entire space-time extent of the noisy data with $C = 4$-fold sub-sampling in space and a training time-step size of $\Delta t = 0.02$. We use a third-order TVD Runge-Kutta method for time integration up to final time $T=1$ during training. With this configuration, STENCIL-NET is able to learn a stable and accurate propagator for the discretized KdV dynamics from the noisy data ${\textbf{V}}$, enabling it to separate data from noise, as shown in Fig. 13.

Also for this noisy case, we compare STENCIL-NET with CNN and FNO architectures, as in the Burgers case without noise. The results in Table 3 show that for low sub-sampling factors, the CNN better predicts the ground-truth signal than the STENCIL-NET, but the STENCIL-NET generalizes significantly better to coarser grids. However, we were unable to train a stable STENCIL-NET with 2264 parameters (used on $4\times$ and $8\times$ downsampling) for prediction with $2\times$ downsampling. This difference in parameterization could cause the spectral properties of the neural networks to differ, such that error estimates cannot be compared across resolutions in this case. Like in the forced Burgers case, the STENCIL-NET is computationally more efficient than both the CNN and FNO (Table 4). Taken together, this example shows the potentially powerful capabilities of STENCIL-NET to extract consistent dynamics from noisy data.

**Table 3﻿:** Prediction Mean Square Error (MSE) comparison for the Korteweg-de Vries (KdV) test case with different spatial sub-sampling and $q=4$ after 10,000 epochs of training.

Prediction MSE	$2\times$	$4\times$	$8\times$
STENCIL-NET	0.03276	$\mathbf {0.01024}$	$\mathbf {0.02509}$
CNN	$\mathbf {0.01532}$	0.02174	0.07613
FNO	0.03078	0.04860	0.06978

For $4\times$ and $8\times$ sub-sampling, the STENCIL-NET has 2264 parameter, the CNN 2281, and the FNO 2665. For $2\times$ sub-sampling, the STENCIL-NET has 8897 parameters, the CNN 8761, and the FNO 8681. In all cases, the prediction error is averaged over a time window two times longer than the training domain. MSEs are averaged over all stable configurations from 5 independent repetitions of the experiment for different network initialization.

Best performance is highlighted in bold.

**Table 4:** Top: Average (over 1000 epochs) training time per epoch compared between the STENCIL-NET, CNN, and FNO from Table 3 with $4\times$ spatial sub-sampling for different training time horizons *q*. Bottom: Average (over 50 time steps) simulation/inference time per time-step for the KdV test case with different sub-sampling. Best performance is highlighted in bold as measured on a Nvidia A100-SXM4 GPU.

Training time (sec)	$q=2$	$q=4$	$q=6$
STENCIL-NET	$\mathbf {0.01395}$	$\mathbf {0.02582}$	$\mathbf {0.03770}$
CNN	0.03880	0.07572	0.1132
FNO	0.06878	0.1284	0.1902

Simulation time (sec)	$2\times$	$4\times$	$8\times$
STENCIL-NET	$\mathbf {0.00120}$	$\mathbf {0.00119}$	$\mathbf {0.00111}$
CNN	0.00312	0.00307	0.00303
FNO	0.00640	0.00600	0.00559

## Conclusions

We have presented the STENCIL-NET architecture for equation-free forecasting from data. STENCIL-NET uses patch-wise parametric pooling and discrete time integration constraints to learn propagators of the discrete dynamics on multiple resolution levels. The design of the STENCIL-NET architecture rests on a formal connection between MLP convolutional layer, rational functions, and solution-adaptive WENO finite-difference schemes. This renders STENCIL-NET approximations valid numerical discretizations of some latent nonlinear dynamics. The accuracy of the predictions also translates to better generalization power and extrapolation stability to coarser resolutions than both Convolutional Neural Networks (CNNs) and Fourier Neural Operators (FNOs), while being computationally more efficient in both training and inference/simulation. Through spectral analysis, we also found that neural architectures capture the power spectrum of the true dynamics, discounting the effects of spectral bias when checking for consistency. We have thus shown that STENCIL-NET can be used as a fast and accurate forecaster of nonlinear dynamics, for model-free autonomous prediction of chaotic dynamics, and for detecting latent dynamics in noisy data.

STENCIL-NET provides a general template for learning representations conditioned on discretized numerical operators in space and time. It combines the expression power of neural networks with the inductive bias enforced from numerical time-stepping, leveraging the two for stable and accurate data-driven forecasting. Beyond being a fast and accurate extrapolator or surrogate model, achieving three to four orders of magnitude speedup over traditional numerical solvers for cases where governing equations are known, a STENCIL-NET can be repurposed to learn closure corrections in computational fluid dynamics and in active material models. Along the same lines, a STENCIL-NET can also be repurposed to learn corrections in coarse-grained discretizations using existing numerical methods (e.g., spectral methods or finite-volume methods) in a hybrid machine-learning/simulation workflow. Finally, since STENCIL-NET is able to directly operate on noisy data, as we have shown here, it can also be used to decompose spatiotemporal dynamics from “propagator-free” noise in application areas such as biology, neuroscience, and finance, where physical models of the true dynamics may not be available.

Future work includes extensions of the STENCIL-NET architecture to 2D and 3D problems over time and to delayed coordinates for inferring latent variables. In addition, combined learning of local and global stencils could be explored.

In the interest of reproducibility, we publish our GPU and multi-core CPU Python implementation of STENCIL-NET and also make all of our trained models and raw training data available to users. They are available from https://github.com/mosaic-group/STENCIL-NET.

## Data Availability

The source code, trained models, and data reported in this study are available at: https://github.com/mosaic-group/STENCIL-NET.
